# Impact of Affordable Care Act Provisions on the Racial Makeup of Patients Enrolled at a Deep South, High-Risk Breast Cancer Clinic

**DOI:** 10.1007/s40615-024-02104-y

**Published:** 2024-09-05

**Authors:** Jillian Tinglin, M. Chandler McLeod, Courtney P. Williams, Meghan Tipre, Gabrielle Rocque, Andrew B. Crouse, Helen Krontiras, Lily Gutnik

**Affiliations:** 1 University of Alabama (UAB) Heersink School of Medicine, 1670 University Blvd, Birmingham, AL 35233 USA; 2 UAB Department of Surgery, Birmingham, AL 35233 USA; 3 UAB Department of Medicine, Birmingham, AL 35294 USA; 4 UAB Division of Preventive Medicine, Birmingham, AL 35233 USA; 5 Department of Medicine, University of Pittsburgh, Pittsburgh, PA 15213 USA; 6 Division of Hematology and Oncology, UAB, Birmingham, AL 35233 USA; 7 UAB Hugh Kaul Precision Medicine Institute, Birmingham, AL 35294 USA

**Keywords:** Breast cancer, High-risk, Preventive care, Affordable Care Act, Disparities, African American, Race

## Abstract

**Purpose:**

Black women are less likely to receive screening mammograms, are more likely to develop breast cancer at an earlier age, and more likely to die from breast cancer when compared to White women. Affordable Care Act (ACA) provisions decreased cost sharing for women’s preventive screening, potentially mitigating screening disparities. We examined enrollment of a high-risk screening program before and after ACA implementation stratified by race.

**Methods:**

This retrospective, quasi-experimental study examined the ACA’s impact on patient demographics at a high-risk breast cancer screening clinic from 02/28/2003 to 02/28/2019. Patient demographic data were abstracted from electronic medical records and descriptively compared in the pre- and post-ACA time periods. Interrupted time series (ITS) analysis using Poisson regression assessed yearly clinic enrollment rates by race using incidence rate ratios (IRR) and 95% confidence intervals (CI).

**Results:**

Two thousand seven hundred and sixty-seven patients enrolled in the clinic. On average, patients were 46 years old (SD, ± 12), 82% were commercially insured, and 8% lived in a highly disadvantaged neighborhood. In ITS models accounting for trends over time, prior to ACA implementation, White patient enrollment was stable (IRR 1.01, 95% CI 1.00–1.02) while Black patient enrollment increased at 13% per year (IRR 1.13, 95% CI 1.05–1.22). Compared to the pre-ACA enrollment period, the post-ACA enrollment rate remained unchanged for White patients (IRR 0.99, 95% CI 0.97–1.01) but decreased by 17% per year for Black patients (IRR 0.83, 95% CI 0.74–0.92).

**Conclusion:**

Black patient enrollment decreased at a high-risk breast cancer screening clinic post-ACA compared to the pre-ACA period, indicating a need to identify factors contributing to racial disparities in clinic enrollment.

**Supplementary Information:**

The online version contains supplementary material available at 10.1007/s40615-024-02104-y.

## Introduction

Black women are less likely to undergo breast cancer screening, and more likely to present with more advanced breast cancers at initial mammographic screening when compared to other racial groups [1]. Differences in access to screening services and persistent barriers to the cost of screening including insurance status and socioeconomic status (SES) have been explored as agents contributing to these discrepancies [1]. Women who are at average risk (lifetime risk of 13%) of developing breast cancer are recommended to begin mammographic screening at age 40, whereas women who are considered at high-risk, defined as having a 20% or greater lifetime risk for developing breast cancer based on comprehensive risk assessment or genetic mutations, benefit from additional screening measures and being in a high-risk screening program [2]. Factors such as prior history of breast cancer, age > 35 years with a 5-year Gail model risk > 1.7%, a lifetime risk greater than 20% based on lobular carcinoma in situ (LCIS) or atypical ductal hyperplasia/atypical lobular hyperplasia (ADH/ALH), a lifetime risk greater than 20% based on models emphasizing familial history, being aged 10–30 years with history mantle irradiation, and a familial pedigree with a known genetic condition, characterize the high-risk population as well as confer their lifetime risk [3]. US studies have demonstrated that while the number of newly diagnosed cases of breast cancer remain similar for Black and White women, the incidence of breast cancer before the age of 45 is higher amongst Black women compared to their White counterparts [4]. Additionally, Black women are more likely to die of breast cancer at every age when compared to their White counterparts [4].

Uptake of clinical preventive services has been historically low among high-risk women, and further aggravated by racial disparities [2, 5]. Although considerable studies have demonstrated the effectiveness of risk-reducing strategies such as tamoxifen, raloxifene, anastrozole, and prophylactic bilateral risk-reducing mastectomy in the high-risk population, their clinical benefit is hinged on the widespread acceptance and use by the high-risk community [6, 7]. For example, though chemoprevention medications have been shown to decrease future breast cancer risk by as much as 50%, its uptake remains low, with less than 50% of eligible patients using it [6, 8]. Although undergoing bilateral risk-reducing mastectomy can decrease breast cancer risk up to 95%, uptake also remains at less than 40% [9, 10]. Insurance status is a notable factor driving racial differences in access to recommendation for genetic counseling and testing, an important step in establishing one’s status as high-risk and thus eligibility for participation in a high-risk screening program [11]. Clinical trials examining chemoprevention use have been historically lacking in Black enrollment. Data from the STAR trial and other studies demonstrated the need for increased provider education to facilitate Black patients’ comprehension about their risk assessment and high-risk status [12, 13]. This educational disparity among Black patients was highlighted in communities where there are fewer high-risk clinics available and more robust risk counseling strategies should be incorporated [12]. Studies have also found that while Black women may be aware of their high-risk status, they are overall less aware of chemoprevention as a treatment option compared to White women [14]. Disparities in insurance status, access to high-risk clinics within communities of color, education regarding breast cancer risk, and overall awareness of risk-reducing therapy have further driven these differences in uptake of preventive services in the high-risk community between Black and White women.

Defined by the five basic tenets of availability, accessibility, accommodations, affordability, and acceptability, access is one common barrier for health disparities existing at the state and national level [15]. To improve access to health care, the Affordable Care Act (ACA) was a landmark bill that increased the overall percentage of insured individuals in the US. It also aimed to improve access to preventive services by decreasing cost sharing for insured women, including decreased cost for screening mammograms every 2 years for women over the age of 40 [16]. Studies have shown increased rates in the uptake of breast cancer screening services since the passage of the ACA as facilitated by increased access to insurance coverage via Medicaid expansion for lower income individuals [17]. Although many studies show that overall breast cancer screening rates increased in Medicaid expansion states, non-expansion states also experienced increased uptake of breast cancer screening services [18, 19]. In the context of non-expansions states like Alabama where higher levels of poverty exist amongst its Black residents, the ACA may have influenced an increased uptake of women’s preventive services in the post-expansion period [20].

Considering the potential impact of the ACA on increasing affordability and therefore accessibility to women’s preventive screening, especially in states where higher numbers of Black residents live in poverty, we evaluated the change in clinic enrollment and racial make-up at the University of Alabama at Birmingham’s Preventive Care Program for Women’s Cancer (UABPC) before and after ACA implementation. The UABPC is a high-risk women’s cancer prevention clinic founded in 2001, housed in UAB’s Comprehensive Cancer Center, a tertiary academic medical center. Patients are referred to this clinic by providers (i.e., primary care physicians or gynecologists) or are self-referred if they are perceived to be potentially at high-risk for developing breast cancer (i.e., 20% or higher based on risk calculators, high-risk breast pathology like atypia, or genetic mutations). The providers at UABPC assess these patients and first confirm that they are truly deemed high-risk. If eligible and confirmed to be high-risk, patients enroll in the clinic and are monitored with risk-reducing management strategies including, genetic counseling, physical examinations, mammograms, ultrasound and breast MRI, chemoprevention therapy, prophylactic surgery with and without reconstruction, and education surrounding lifestyle modifications that can positively influence breast health. Following their initial referral, patients are seen annually to monitor their lifetime risk for breast cancer and continually tailor their risk-reducing management strategy. The UABPC is the first clinic of its kind in Alabama, a state in which the incidence of breast cancer is higher than the national average, and Black women are more likely to die at an increased rate compared to the national average. Given this, we hypothesized that there would be an increase in the rate of Black patient enrollment within the UABPC post-ACA when compared to pre-ACA levels.

## Methods

### Study Design and Patient Population

This retrospective, quasi-experimental study used an interrupted time series approach to examine the impact of ACA provisions upon the racial makeup of the UABPC. Women aged 20–82 years seen for their initial UABPC appointments between February 2003 and February 2019 who consented to be in the UABPC database were included in this study. Appointments were scheduled through patients referred internally through UAB clinics, external non-UAB clinics, as well as through self-referral. Exclusion criteria included primary residence outside of Alabama. IRB approval was subsequently obtained (IRB-030409006).

### Outcomes: Racial Composition of Patients Seen at the UABPC

The primary outcome for this study was change in the racial composition of patients seen at the UABPC clinic pre- versus post-implementation of ACA provisions. Racial composition of women seen at the UABPC was calculated using the count of (1) White, or (2) black or African American patients seen in comparison relation to all patients seen at the UABPC by month. Counts of Asian, Hispanic, other, and unknown categories were not evaluated in the ITS analysis estimates due to small sample sizes. However, they were still accounted for when calculating monthly proportions of White and Black enrollments.

### Exposure: Implementation of ACA Provisions

The exposure of interest was implementation of ACA provisions, effective January 1, 2014. We examined monthly pre-intervention time periods prior to ACA implementation (February 2003 to December 2013) monthly post-intervention time periods (January 2014 to February 2019).

### Covariates

Patient sociodemographics abstracted from the UABPC database and electronic health record included age at enrollment, race (White, Black, or African American, other [Hispanic, Native American, unknown]), insurance status (private/commercial, Medicaid, Medicare, other [self-pay, Tricare, charity care, other]), and home address. Patient addresses were used to identify neighborhood disadvantage on the county level, as measured by the Area Deprivation Index (ADI). ADI was scored from 1 to 100%, with higher percentages representing higher neighborhood disadvantage. For this study, Alabama census tract ADI values for 2015 and 2020 were averaged by county and then linked to patients’ records using patient county and enrollment date. Patients who enrolled before 2017 were assigned their county’s mean 2015 ADI and patients who enrolled in 2017 or later were linked to their county mean 2020 ADI. ADI was dichotomized into high (> 85%) and low (≤ 85%) neighborhood disadvantage [21].

### Analysis

Descriptive statistics, including means and standard deviations for continuous variables and frequencies for categorical variables, were calculated for patient sociodemographic data and compared pre- and post-ACA implementation. Bivariate comparisons utilized Student’s *t*-test and chi-squared test for continuous and categorical variables, respectively. An interrupted time series analysis using a quasi-Poisson model with log link and an offset for the log of the total number of enrollments was used to assess our primary outcome using the following level-change regression model [22]:

$$\mathrm{Log}\;\left(Yt\right)==\log\;\left(n\;{\mathrm{total}}_{\mathit t}\right)+\beta_0+\beta_1\ast\;\mathrm{time}\;+\beta_2\ast\;(\mathrm{ACA}\operatorname{implementation})+\beta_3\ast\;(\mathrm{time}\ast\mathrm{ACA}\operatorname{implementation})$$

In the described basic model, the coefficient *β*_0_ estimates the expected log number of patients in Q1 2003 among all patients, and *β*_1_ estimates the trend of log number of patients seen per year prior to ACA implementation. The coefficient *β*_2_ is the immediate effect of ACA provisions on the log number of patients enrolled after the intervention, and *β*_3_ is the change in the trend of the log number of patients seen per year post-ACA implementation relative to the pre-ACA period. The offset consists of the natural log of the total number of enrollments for the time period. While enrollments were aggregated on a quarterly basis, coefficients were evaluated for 1-year change. Exponentiation of coefficients provides for the corresponding estimated incident rate ratios. Separate models were constructed to assess enrollment rates for White and Black or African American patients. To account for potential confounding by socioeconomic status, sensitivity analyses were performed for each model adjusting for neighborhood disadvantage as measured by ADI. ADI was incorporated into models by calculating the measure of patients who had ADI values greeter than 85% in each quarter. Analysis was performed in R (version 4.2.1, 2022) and significance was assessed at *p*-value < 0.05.

## Results

### Study Sample

**Fig. 1:**
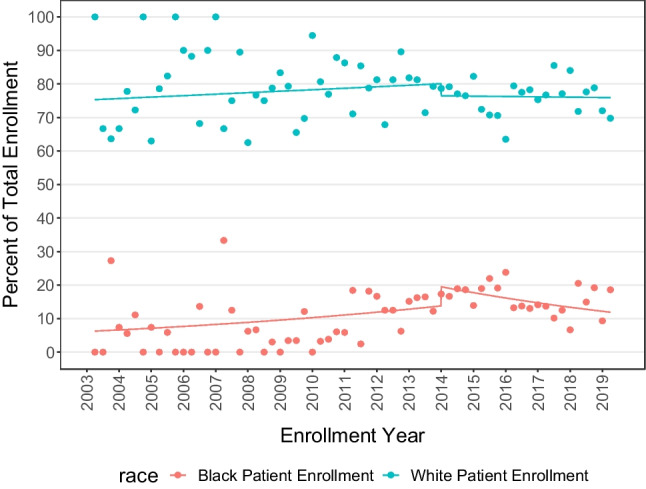
Percentage of Black and White Enrollment to UAB Preventive Care Program for Women’s Cancer by race from Q1 2003 to Q1 2019. Points indicate observed quarterly values and solid lines indicate predicted values from fitted model

Of the 2767 women seen at the UABPC, mean age was 46 years (SD 13), 13% were Black or African American, 82% were commercially insured, and 8% lived in a highly disadvantaged neighborhood (Table 1 and Fig. 1). Compared to the time period before ACA, patients seen after implementation of ACA provisions were younger (mean age 45 vs. 48).

**Table 1:** Patient sociodemographic characteristics by time seen at the UAB Preventive Care Program for Women’s Cancer (*N* = 2737)

	Total *N* (%) (*N* = 2767)	Pre-Affordable Care Act Provisions *N* (%) (*n* = 1285)	Post-Affordable Care Act Provisions *N* (%) (*n* = 1 482)	*p*-value
Age (mean, SD)	46 (13)	48 (12)	45 (13)	.001
Race				< .001
Black/African American	362 (13)	129 (10)	233 (16)	
White	2134 (78)	1006 (78)	1128 (77)	
Other^*^	248 (9)	147 (12)	101 (7)	
Insurance status				.16
Commercial	2172 (82.1)	944 (80.6)	1228 (83.4)	
Medicare	312 (11.8)	146 (12.5)	166 (11.8)	
Medicaid	56 (2.1)	26 (2.2)	30 (2.0)	
Other**	104 (3.7)	55 (4.3)	49 (3.3)	
Missing***	123	114	9	
Neighborhood disadvantage status				.28
High disadvantage (ADI > 85)	132 (8.3)	64 (7.7)	68 (9.0)	
Low disadvantage (ADI < 85)	1452 (91.7)	763 (92.3)	689 (91.0)	
Missing	1183	458	725	

*SD* standard deviation

*Asian, Native American, Hispanic, other, unknown

**Self-pay, Tricare, Charity, Agency

***Not included in *p*-value calculation

### Racial Trends for Patients Seen at the UABPC

ITS analysis estimates indicated rates of White patient enrollment at the UABPC did not increase or decrease during the pre- (IRR 1.01 95% CI 1.00–1.02) or post-ACA implementation periods (IRR 1.00 95% CI 0.98–1.02) (Table 2). Conversely, rates of Black patient enrollment increased by 13% per year pre-ACA implementation (IRR 1.13, 95% CI 1.05–1.22). Immediately, post-ACA implementation, the rate of White and Black patient enrollment did not change significantly (IRR 1.34, 95% CI 0.93–1.94 and IRR 0.96, 95% CI 0.89–1.03). However, post-ACA implementation, rate of Black patient enrollment decreased by 17% per year (IRR 0.83, 95% CI 0.74–0.92) compared to the pre-ACA rate of Black patient enrollment. In sensitivity analyses adjusting for neighborhood disadvantage in the ITS analysis, similar trends were observed (Supplemental Table 1).

**Table 2:** Interrupted time series quasi-Poisson model-estimated rates of UAB Preventive Care Program for Women’s Cancer enrollment by White and Black race (*n* = 65 quarters)

	Rate of White enrollment (comparing change in rate from pre-ADA to post-ADA)			Rate of White patient enrollment (evaluating change in rate in pre and post ADA periods)			Rate of Black/African American enrollment (comparing change in rate from pre-ADA to post-ADA)			Rate of Black/African American patient enrollment (evaluating change in rate in pre and post ADA periods)		
	IRR	95% CI	*p*	IRR	95% CI	*p*	IRR	95% CI	*p*	IRR	95% CI	*p*
Time (year)	1.01	1.00–1.02	0.27	1.01	1.00–1.02	0.27	1.13	1.05–1.22	< 0.01	1.13	1.05–1.22	< 0.01
ACA initiation	0.96	0.89–1.03	0.27	0.96	0.89–1.03	0.27	1.34	0.93–1.94	0.11	1.34	0.93–1.94	0.11
Change in rate of patients enrolled per year after ACA implementation	0.99	0.97–1.01	0.37	1.00	0.98–1.02	0.85	0.83	0.74–0.92	0.001	0.93	0.86–1.01	0.10

*IRR* incidence rate ratio, *CI* confidence interval

## Discussion

In the 5-year period following ACA implementation, Medicaid expansion and implementation of cost-sharing preventive services for average risk women in the US, yearly rates of Black and White patient enrollment remained stable. Average risk women aged 40–74 are recommended to obtain routine annual or biennial mammography to detect breast cancers at earlier stages to decrease breast cancer–associated deaths [23]. Studies have shown that routine mammography screening can decrease risk of death from breast cancer by up to 22%. [24]. As a result of these recommendations, breast cancer mortality rates have notably decreased by 40% since 1989 [25]. Following the passage of the ACA, certain provisions further increased patient access to routine mammogram screening, potentially improving breast cancer mortality risk. According to the National Cancer Institute, the average percentage of average risk Black women receiving routine mammograms increased from 73 to 75% in the pre- vs. post-ACA time period, and the average percentage of average risk White women receiving routine mammograms remained stable at 74% throughout the pre- and post-ACA time period [26]. Mammography remains the most cost-effective and clinically impactful form of screening for decreasing the breast cancer mortality rate; however, additional screening and diagnostic measures are considered for women at higher risk [27].

Patients determined to be at high-risk should explore further screening options to mitigate their lifetime risk [3]. Given that 1% of all women in the US are classified as high-risk with a higher incidence of breast cancer within the community, there is considerable potential to decrease their mortality risk through continued identification, surveillance, and screening [28, 29]. Approximately 5–10% of all breast cancers are inherited [30]. Of those heritable cancers, 30% are caused by germline BRCA1/2 mutations [30]. Risk management begins with risk stratification which includes gathering further information regarding genetic testing, familial, personal, and reproductive history [3]. Currently used risk assessment models include BRCAPRO, the Yale University model, International Breast Cancer Intervention Study (IBIS)/Tyrer-Cruzik model, and the Breast and Ovarian Analysis of Disease Incidence and Carrier Estimation Algorithm (BOADICEA) [31]. While these models are widely accepted and utilized, they each incorporate a variety of factors that determine risk [32]. These models, as well as average risk screening recommendations were developed and validated using a predominantly White patient sample [33, 34]. The Gail model was one of the first and most widely accepted risk assessment models used; however, it was not validated for use in minority populations since the original case study on which it was based sampled 200,000 White women only [35, 36]. Though later updates to the Gail model incorporated a more diverse patient sample from the CARE study, it was still found to significantly underestimate breast cancer risk in Black women [37]. More recently, the Black Women’s Health Study (BWHS) model was developed to estimate breast cancer risk in Black women, in effort to mitigate the existing underrepresentation in current models [36]. In the context of the effect of ACA implementation on the rate of patient enrollment relative to the pre-ACA period for the UABPC high-risk breast cancer screening clinic, our results showed that rates of White patient enrollment remained consistent throughout pre- and post-ACA implementation periods. Rates of Black patient enrollment, however, decreased in the post-ACA time period relative to the pre-ACA time period.

Further research must be done to elucidate the factors associated with the significant decrease in Black patient enrollment following implementation of expanded women’s preventive services by the ACA compared to the pre-ACA implementation period. Studies have shown that Black women are less likely to obtain genetic testing that would establish their high-risk status, and are 25% less likely to be referred by physicians to obtain additional testing after being established as high-risk when compared to White women [38, 39]. Access to adequate insurance and insurance status may have also attributed to changes in Black patient enrollment. Since Alabama was a non-Medicaid expansion state, ACA implementation followed by Medicaid expansion could have resulted in women seeking care elsewhere due to increased access to other screening options. Even with increased access through the ACA, studies have also demonstrated that physicians referred over 20% more high-risk White patients on Medicaid to high-risk services when compared to Black patients [38]. Racial differences in access to comprehensive care and high-risk screening modalities such as breast MRI are multifactorial and rooted in historical scientific underrepresentation of Black women and other minorities [40].

The UABPC is unique in that it is the only dedicated high-risk prevention clinic in Alabama, though women can be managed with high-risk screening strategies in other settings, for example by their primary care physicians or gynecologists [41, 42]. However, while 71% of primary care providers (PCPs) report obtaining a family history during routine visits, less than half reported discussing pertinent aspects of patients’ history as it pertains to their breast cancer risk such as history of breast biopsies, obstetrical history, or age at menarche [43]. Only 3% of PCPs stated they calculated Gail model risk scores, 76% reported never having calculated a breast cancer risk assessment score, and 50% stated they were unaware of what the Gail model is used for [43]. Another study found that obstetrician gynecologists (OB/GYNs), while more likely to conduct a breast cancer risk assessment than their internist or family practitioner colleagues, only 29% of OB/GYNs reported using risk assessment models for their patients and 43% reported ordering testing for BRCA 1/2 mutations [44]. Thus, the UABPC presents a unique opportunity to study this population as a surrogate for understanding potential issues surrounding access and disparities in high-risk patients. Publicization of high-risk screening programs like the UABPC and its referral process may be optimized to improve access, given low rates of comprehensive risk assessment and management of high-risk patients in the community setting [43, 44]. For example, knowledge of the UABPC is largely spread through internal providers within the UAB healthcare system by word of mouth rather than a standardized referral process. Providers external to the UAB system are less likely to be aware of the clinic, and thus less likely to refer patients. Additionally, many patients are self-referrals and are aware of the clinic via spoken communication. This is another opportunity to examine racial disparities, as these referral patterns can be evident in other similar settings with limited dedicated high-risk screening clinics.

ACA implementation and Medicaid expansion was enacted as a path to increase the percentage of the insured in the US, especially those in low-income communities. Historically, more African Americans live in poverty or at a lower socioeconomic status when compared to their White counterparts [45]. Although the ACA was targeted at this community and other communities at lower socioeconomic status, we noted a decrease in the enrollment of Black patients during the post-expansion period. According to a study utilizing data from the Behavioral Risk Factor Surveillance System (BRFSS) between 2012 and 2016, screening among average risk, low-income women increased significantly by 4.9% in expansion and non-expansion states [46]. Additional studies examining the post-ACA period (2013–2014) found that compared to pre-ACA, there was an increased incidence in the detection of early-stage breast cancer in the average risk population from 55.5 to 56.9 cases per 100,000 person-years, which was consistent with increased trends following ACA passage [47]. We are unable to find studies reflecting the impact of the ACA on the high-risk community, but given the aforementioned data believe it is reasonable to surmise these findings would follow similar trends in the high-risk population.

In exploring the potential factors for this downward trend in enrollment, it is possible that the historical distrust of the healthcare system within the Black community could have adversely influenced Black patient enrollment in the UABPC. This pervasive and warranted distrust is vested in the historical mistreatment and abuse of African Americans by the US healthcare system, with levels of mistrust higher amongst those African Americans that are known descendants of the enslaved [48]. In certain minority communities, health concerns and hardships are sometimes considered culturally taboo, and therefore it can be difficult to encourage patients to seek out a primary care physician and in turn receive necessary referral to high-risk clinic like the UABPC that is equipped to screen them appropriately [49]. During the post-ACA period, it is possible there may have been a decrease in providers in the surrounding area, leading to a decreased access to care. It can be equally challenging for a patient who is already distrustful of the healthcare system to reestablish care with a provider with whom they do not have a well-developed relationship. Because of these potential environmental shifts that can contribute to patient care and could cause a downtrend in UABPC patient enrollment, further investigation is warranted to understand the referral process to the UABPC and communities it predominantly serves.

Uninsurance or underinsurance is associated with other social determinants of health such as being a racial minority, having a lower educational status, or earning a lower wage [50, 51]. These characteristics predispose patients to a decreased potential of upward social mobility, influencing their overall socioeconomic status and directly obstructing access to preventive care or appropriate screening for their high-risk status. Although non-Medicaid expansion states like Alabama did not receive additional funding to expand women’s preventive services, many non-Medicaid expansion states still experienced an increase in insurance utilization following Medicaid-expansion, likely due to the increased awareness of insurance accessibility and importance of preventive healthcare initiatives popularized by the ACA [50]. If Alabama were to expand Medicaid, its citizens could experience increased insurance access and access to breast cancer screening modalities, incur lower incidences of late-stage breast cancer diagnoses, and become a more health-informed population [52].

A notable limitation in this study was that the initial provisions within the ACA decreased cost sharing for women’s preventive services for women at average risk for developing breast cancer, rather than high-risk. Despite this, we felt trends reflected in the average risk community, such as increased mammography screening, would be mirrored in the high-risk community. In addition to this limitation, we acknowledge that further investigation into the clinic referral patterns of the UABPC may yield a better understanding of the decreased enrollment of Black patients in the UABPC during the post-ACA period. Additionally, our study reflects the trends of enrollment for those patients who were able to access the UABPC either through self or physician referral. Therefore, our trends may not be incorporating those patients that are the most under-represented which would suggest a more notable inequity in access to high-risk preventive services in the post-expansion period.

## Conclusions

Our data demonstrated there was a statistically significant decrease in Black patient enrollment rate in the post-ACA time period relative to the pre-ACA time period in a dedicated high-risk screening program. Racial disparities persist despite nationwide efforts to improve access to preventive healthcare, as is demonstrated in our study within a unique clinic.

Moving forward, we plan to continue exploring the persistent disparities in breast cancer amongst communities of color following the passage of the ACA. In addition to increased publicization of preventive care clinics such as the UABPC as well as a greater understanding of the referral process of the UABPC, in the future we plan to expand our area of research to incorporate other high-risk populations in the Southeast US, incorporating other comorbidities and chronic illnesses impacting communities of color and possibly dictating their access to care. Multi-level interventions addressing racial disparities in the high-risk breast cancer community are needed to increase equity for access of preventive services.

## Supplementary Information

Below is the link to the electronic supplementary material.

Supplementary file1 (DOCX 89 KB)

## Data Availability

Patient data was housed and pulled from the UABPC RedCap database and UABPC Electronic Health Record (EHR).
