# Supplementary material for: Impact of Affordable Care Act Provisions on the Racial Makeup of Patients Enrolled at a Deep South, High-Risk Breast Cancer Clinic
Source: J Racial Ethn Health Disparities. 2024 Sep 5;12(5):2965–73. doi: 10.1007/s40615-024-02104-y (PMC11880353; doi:10.1007/s40615-024-02104-y)
Supplement: Supplementary file 1 — Supplementary file1 (DOCX 89 KB) [file 40615_2024_2104_MOESM1_ESM.docx]

**Supplementary Table 1**. Interrupted time series quasi-Poisson model-estimated rates of UAB Preventive Care Program for Women’s Cancer enrollment by White (n = 65 quarters with ADI information), and Black race (n = 52 quarters with ADI information), adjusted for ADI.

|  | **Rate of White**  **patient enrollment**  **adjusted by ADI** | | | **Rate of Black/African American patient enrollment**  **adjusted by ADI** | | |
| --- | --- | --- | --- | --- | --- | --- |
|  | IRR | 95% CI | *p* | IRR | 95% CI | *p* |
| Time (year) | 1.01 | 1.00-1.02 | 0.3 | 1.08 | 1.01-1.15 | 0.03 |
| ACA initiation | 0.95 | 0.89-1.03 | 0.21 | 1.41 | 1.03-1.95 | 0.04 |
| Change in rate of patients enrolled per year after ACA implementation | 0.99 | 0.97-1.01 | 0.5 | 0.85 | 0.76-0.94 | <0.01 |
| Patients with ADI>85% | 0.76 | 0.49-1.20 | 0.24 | 1.89 | 1.15-3.07 | 0.01 |

IRR=incidence rate ratio; CI=confidence interval

\

Supplementary Table 2: Number of Black and White patients enrolled per quarter.

| Enrollment  Quarter | Black Patient Enrollment | White Patient Enrollment | Total Enrollment |
| --- | --- | --- | --- |
| 03-Q1 | 0 | 1 | 1 |
| 03-Q2 | 0 | 2 | 3 |
| 03-Q3 | 3 | 7 | 11 |
| 03-Q4 | 2 | 18 | 27 |
| 04-Q1 | 1 | 14 | 18 |
| 04-Q2 | 2 | 13 | 18 |
| 04-Q3 | 0 | 3 | 3 |
| 04-Q4 | 2 | 17 | 27 |
| 05-Q1 | 0 | 11 | 14 |
| 05-Q2 | 1 | 14 | 17 |
| 05-Q3 | 0 | 5 | 5 |
| 05-Q4 | 0 | 9 | 10 |
| 06-Q1 | 0 | 15 | 17 |
| 06-Q2 | 3 | 15 | 22 |
| 06-Q3 | 0 | 9 | 10 |
| 06-Q4 | 0 | 8 | 8 |
| 07-Q1 | 1 | 2 | 3 |
| 07-Q2 | 1 | 6 | 8 |
| 07-Q3 | 0 | 17 | 19 |
| 07-Q4 | 1 | 10 | 16 |
| 08-Q1 | 2 | 23 | 30 |
| 08-Q2 | 0 | 15 | 20 |
| 08-Q3 | 1 | 26 | 33 |
| 08-Q4 | 0 | 5 | 6 |
| 09-Q1 | 1 | 23 | 29 |
| 09-Q2 | 1 | 19 | 29 |
| 09-Q3 | 4 | 23 | 33 |
| 09-Q4 | 0 | 17 | 18 |
| 10-Q1 | 1 | 25 | 31 |
| 10-Q2 | 1 | 20 | 26 |
| 10-Q3 | 2 | 29 | 33 |
| 10-Q4 | 3 | 44 | 51 |
| 11-Q1 | 7 | 27 | 38 |
| 11-Q2 | 1 | 35 | 41 |
| 11-Q3 | 6 | 26 | 33 |
| 11-Q4 | 8 | 39 | 48 |
| 12-Q1 | 7 | 38 | 56 |
| 12-Q2 | 8 | 52 | 64 |
| 12-Q3 | 3 | 43 | 48 |
| 12-Q4 | 5 | 27 | 33 |
| 13-Q1 | 13 | 65 | 80 |
| 13-Q2 | 15 | 65 | 91 |
| 13-Q3 | 10 | 65 | 82 |
| 13-Q4 | 13 | 59 | 75 |
| 14-Q1 | 12 | 57 | 72 |
| 14-Q2 | 14 | 57 | 74 |
| 14-Q3 | 19 | 78 | 102 |
| 14-Q4 | 11 | 65 | 79 |
| 15-Q1 | 11 | 42 | 58 |
| 15-Q2 | 18 | 58 | 82 |
| 15-Q3 | 13 | 48 | 68 |
| 15-Q4 | 15 | 40 | 63 |
| 16-Q1 | 9 | 54 | 68 |
| 16-Q2 | 11 | 62 | 80 |
| 16-Q3 | 3 | 18 | 23 |
| 16-Q4 | 12 | 64 | 85 |
| 17-Q1 | 10 | 56 | 73 |
| 17-Q2 | 7 | 59 | 69 |
| 17-Q3 | 12 | 74 | 96 |
| 17-Q4 | 5 | 63 | 75 |
| 18-Q1 | 16 | 56 | 78 |
| 18-Q2 | 10 | 52 | 67 |
| 18-Q3 | 10 | 41 | 52 |
| 18-Q4 | 7 | 54 | 75 |
| 19-Q1 | 8 | 30 | 43 |

Supplementary Figure 1: Percentage of Black and White Enrollment to UAB Preventive Care Program for Women’s Cancer by race from, Q1 2003 to Q1 2019, adjusted for ADI. Points indicate observed quarterly values and solid lines indicate predicted values from fitted model.
